# Buprenorphine Prescribing and Challenges Faced Among National Health Service Corps Clinicians

**DOI:** 10.1001/jamanetworkopen.2024.11742

**Published:** 2024-05-17

**Authors:** Kathleen Rowan, Savyasachi V. Shah, Steven Binns, Elizabeth Murphy, Jennifer Satorius, Alina Ghobadi, Daniel Krauss, Carolyn Robbins, Victoria Schoebel, Alana Knudson, Hayden Kepley

**Affiliations:** 1 NORC at the University of Chicago, Bethesda, Maryland; 2 Health Resources and Services Administration Bureau of Health Workforce, Rockville, Maryland

## Abstract

**Importance:**

The National Health Service Corps (NHSC) Loan Repayment Program (LRP) expansion in fiscal year (FY) 2019 intended to improve access to medication for opioid use disorder (MOUD) by adding more clinicians who could prescribe buprenorphine. However, some clinicians still face barriers to prescribing, which may vary between rural and nonrural areas.

**Objective:**

To examine the growth in buprenorphine prescribing by NHSC clinicians for Medicaid beneficiaries during the NHSC LRP expansion and describe the challenges to prescribing that persist in rural and nonrural areas.

**Design, Setting, and Participants:**

This cross-sectional study analyzed preexpansion and postexpansion Medicaid claims data to evaluate the percentage of prescriptions of buprenorphine filled during FY 2017 through 2021. This study also analyzed challenges and barriers to prescribing MOUD between rural and urban areas, using results from annual surveys conducted with NHSC clinicians and sites from FY 2019 through FY 2021.

**Exposure:**

Prescribing of buprenorphine by NHSC clinicians.

**Main Outcomes and Measures:**

The main outcomes were the percentage and number of Medicaid beneficiaries with opioid use disorder (OUD) who filled a prescription for buprenorphine before and after the LRP expansion and the challenges NHSC clinicians and sites faced in providing substance use disorder and OUD services. Survey results were analyzed using descriptive statistics.

**Results:**

During FYs 2017 through 2021, 7828 NHSC clinicians prescribed buprenorphine (standard LRP: mean [SD] age, 38.1 [8.4] years and 4807 females [78.9%]; expansion LRPs: mean [SD] age, 39.4 [8.1] years and 1307 females [75.0%]). A total of 3297 NHSC clinicians and 4732 NHSC sites responded to at least 1 survey question to the 3 surveys. The overall percentage of Medicaid beneficiaries with OUD who filled a prescription for buprenorphine during the first 2.5 years post expansion increased significantly from 18.9% before to 43.7% after expansion (an increase of 123 422 beneficiaries; *P* < .001). The percentage more than doubled among beneficiaries living in areas with a high Social Vulnerability Index score (from 17.0% to 36.7%; an increase of 31 964) and among beneficiaries living in rural areas (from 20.8% to 55.7%; an increase of 45 523). However, 773 of 2140 clinicians (36.1%; 95% CI, 33.6%-38.6%) reported a lack of mental health services to complement medication for OUD treatment, and 290 of 1032 clinicians (28.1%; 95% CI, 24.7%-31.7%) reported that they did not prescribe buprenorphine due to a lack of supervision, mentorship, or peer consultation.

**Conclusions and Relevance:**

These findings suggest that although the X-waiver requirement has been removed and Substance Abuse and Mental Health Services Administration guidelines encourage all eligible clinicians to screen and offer patients with OUD buprenorphine, as permissible by state law, more trained health care workers and improved care coordination for counseling and referral services are needed to support comprehensive OUD treatment.

## Key Points

**Question:**

What is the association between the National Health Service Corps (NHSC) Loan Repayment Program (LRP) expansion and access to medication for opioid use disorder (MOUD), and what challenges to providing MOUD may NHSC clinicians and sites continue to face?

**Findings:**

In this cross-sectional study of Medicaid claims data for 7828 NHSC clinicians and survey data from 3297 clinicians and 4732 sites, at least 123 422 additional Medicaid beneficiaries were provided with MOUD over the first 2.5 years following the LRP expansion. However, 70% of clinicians reported a lack of addiction counseling to accompany treatment with MOUD as well as other staffing shortages.

**Meaning:**

These findings suggest that additional clinicians and training are needed to increase access to comprehensive treatment with MOUD.

## Teaser

This cross-sectional study examines buprenorphine prescribing to Medicaid beneficiaries and associated challenges faced by National Health Services Corps Loan Repayment Program clinicians.

## Introduction

The opioid epidemic in the US continues to claim more than 100 000 lives annually, and in 2021, it accounted for 75% of all overdose deaths.^1^ The total annual number of deaths from opioid overdose has more than doubled between 2015 and 2021, from 52 623 to 107 573.^2^

Buprenorphine is a long-standing, evidence-based treatment for patients with opioid use disorder (OUD), though it may still be underused.^3,4,5,6^ To expand access to medication for OUD (MOUD), the US Congress repealed the X-waiver requirement in December 2022, and the Drug Enforcement Administration (DEA) and Substance Abuse and Mental Health Services Administration issued new guidelines that have increased the flexibility in the training required for prescribing buprenorphine and removed restrictions on the number of patients physicians can treat.^7^ In May 2023, the DEA also extended flexibilities in using telehealth for prescribing buprenorphine that were enacted during the COVID-19 pandemic.^8^

While these changes have the potential to expand treatment for OUD, clinicians still face challenges and barriers to providing MOUD, including a lack of resources for consultation, stigma, and insufficient education and training.^5,9,10^ Access to MOUD in rural areas is also hampered by the shortage of clinicians in medically underserved areas (ie, areas with limited access to health care).^11,12^

To help combat the opioid crisis in underserved areas, the Health Resources and Services Administration (HRSA) expanded the National Health Service Corps (NHSC) Loan Repayment Program (LRP) to include 2 new programs, the Substance Use Disorder (SUD) LRP and Rural Community LRP (referred to as expansion LRPs in this study), in addition to the existing standard LRP. The NHSC LRP offers primary care, dental, and behavioral health care clinicians the opportunity to have their student loans repaid while working at NHSC sites, which are health care facilities located in underserved communities. These 2 expansion programs emphasize the recruitment of clinicians trained in SUD treatment, whereas the standard program is targeted more for primary care clinicians. The expansion programs require a longer commitment of 3 vs 2 years compared with the standard program, and clinicians in the SUD and Rural Community LRPs receive higher levels of loan payment ($75 000 and $100 000, respectively, compared with $50 000 in the standard program).

Prior work,^13^ conducted as part of an independent evaluation of HRSA’s SUD investments, described the role of the NHSC in expanding behavioral health services in socially vulnerable and rural areas but did not address the expansion of buprenorphine for treatment of OUD or challenges clinicians faced in providing OUD treatment. This prior analysis, conducted with fiscal year (FY) 2019 NHSC clinicians (the first year of the expansion programs), showed that the percentage of NHSC clinicians working in rural areas increased by 59% between FY 2017 and FY 2020. It also showed that 66% (962 of 1562) of NHSC sites added or expanded MOUD, including buprenorphine, methadone, and naltrexone.

The literature on strategies to improve substance use treatment in rural and underserved areas is limited.^14^ As OUD continues to be a substantial public health problem that affects families and communities across the country, addressing persistent challenges through policy options and actions at the state and federal levels may help to mitigate the opioid and substance use epidemic. Thus, the aims of this study are to analyze buprenorphine prescribing for Medicaid beneficiaries, particularly those living in rural and socially vulnerable communities, by NHSC clinicians since the LRP expansion and to describe the challenges and barriers to OUD treatment that may persist since removal of the DEA waiver requirement.

## Methods

### Study Population

In this cross-sectional study, which is part of the larger evaluation,^13^ the analytic population consisted of new NHSC clinicians (new LRP awardees in FY 2019 through FY 2021) and all NHSC sites (FY 2019 through FY 2021). All new clinicians and sites were eligible to participate in the evaluation surveys. The claims analysis was conducted on a subset of the NHSC clinicians who were physicians, nurse practitioners, and physician assistants who prescribed buprenorphine to Medicaid beneficiaries. The National Opinion Research Center’s institutional review board reviewed all aspects of the study’s data collection and determined the study not to be human participants research under Federalwide Assurance No. FWA00000142. The use of clinician and beneficiary claims data complied with a data use agreement through the Centers for Medicare & Medicaid Services Research Data Assistance Center to ensure privacy. Evaluation survey respondents were shown a screen stating that by starting the survey, they agreed to participate in the survey. This study follows the Strengthening the Reporting of Observational Studies in Epidemiology (STROBE) reporting guideline for cross-sectional studies.

We used clinicians’ National Provider Identifiers (NPIs), available from the NHSC application data obtained from HRSA, to identify them in claims data in the preexpansion and postexpansion periods. Assignment to the standard or expansion groups in these analyses was determined by which 1 of the 3 programs NHSC clinicians selected on their applications. Application data collected included self-reported demographic measures of gender (female, male, and does not wish to disclose), age, underrepresented minority status as reported by HRSA (includes individuals identifying as American Indian or Alaska Native, Black or African American, Native Hawaiian or Other Pacific Islander, and Hispanic [all races]),^15^ and program assignment (standard, SUD, or Rural Community LRP).

For the claims analyses, we grouped clinicians in the 2 expansion programs (SUD and Rural Community) together to enable a comparison to the standard program. We also used 3 years of pooled cross-sectional data from surveys (fielded by the authors) on NHSC clinicians and sites. Survey respondents included NHSC clinicians and sites who answered at least 1 question examined in this study.

### Claims Data

Transformed Medicaid Statistical Information System data were used to determine the percentage and number of beneficiaries with OUD who filled a prescription. Almost 40% of adults aged 18 to 64 years with OUD have health care through Medicaid.^16^ Claims were analyzed for the 2.5 years before (January 1, 2017, to June 30, 2019) and after (July 1, 2019, to December 31, 2021) the NHSC program expansion. The algorithm for medication-assisted treatment from the *Medicaid Section 1115 Substance Use Disorder Demonstrations: Technical Specifications for Monitoring Metrics*, version 4.0 manual^17^ was used to identify beneficiaries with OUD and a prescription. This algorithm includes all beneficiaries with an OUD diagnosis who were enrolled in Medicaid for any amount of time during the measurement period as the denominator. Beneficiaries were classified as having OUD if they had at least 1 claim with a diagnosis code listed under the Healthcare Effectiveness Data and Information Set 2019 Opioid Abuse and Dependence Value Set in the past 12 months (complete list provided in eMethods 1 in Supplement 1). We reviewed the quality of the Medicaid prescription claims using the Medicaid Data Quality Atlas; each topic of prescription claims received a quality rating (low, medium, high, or unusable).^18^ We used data from all states in all years, acknowledging that data for 2 states would be undercounted (Florida and Maine) due to a high level of incompleteness or missing a prescribing NPI (details provided in eMethods 2 in Supplement 1). For claims with a missing prescribing date (approximately 6.8%), we used the fill date because we observed that for prescriptions with both dates, the mean (SD) time between prescription and fill was 3.5 (11.7) days. We included all forms of prescription buprenorphine (eg, alone or in combination with naloxone).

### Rural and Social Vulnerability Data

To understand changes in buprenorphine use for beneficiaries living in rural areas and areas with high social vulnerability, we used county-level rural classification data from the Federal Office of Rural Health Policy to identify beneficiaries living in rural areas.^19^ We also referred to HRSA’s definition of rural as all nonmetropolitan counties, all metropolitan census tracts with Rural Urban Commuting Area codes 4 to 10, and large-area metropolitan census tracts of at least 400 square miles in areas with a population density of 35 or less per square mile with Rural Urban Commuting Area codes 2 to 3.^20^ We used county-level Social Vulnerability Index (SVI) data from the Centers for Disease Control and Prevention’s Agency for Toxic Substances and Disease Registry to identify and classify counties in the highest quintile of SVI scores, indicating the greatest social vulnerability.^21^

### Survey Data

We conducted 3 cross-sectional surveys of new NHSC clinicians (new LRP awardees in FY 2019 through FY 2021) and NHSC sites (FY 2019 through FY 2021) in the fall of each year during 2020 to 2022. Both the NHSC clinician and site surveys included questions about challenges to providing SUD and OUD services. With the organizations’ institutional review board approval, we used NHSC administrative data to identify clinicians and sites in rural areas. The surveys and related materials were approved by the US Office of Management and Budget, and links to the survey materials and more details on the survey development and fielding are provided in eMethods 3 in Supplement 1.

### Statistical Analysis

We provide descriptive statistics on demographic characteristics of clinicians in the analysis. Demographic measures are reported separately for clinicians in the claims analyses (which are stratified by standard and expansion programs) and for clinicians in the survey analyses (which are stratified by rural and nonrural areas). We provide characteristics for clinicians in the survey analyses by standard and expansion programs in eTable 1 in Supplement 1.

We calculated the percentage and number of total Medicaid beneficiaries with OUD with a prescription for buprenorphine in the preexpansion and postexpansion period from claims data. National Provider Identifiers were used to identify NHSC clinicians in the preexpansion and postexpansion period. To test whether the number of beneficiaries increased significantly more among clinicians who joined the expansion LRPs compared with the standard LRP, a difference-in-differences framework was used, with an interaction term for the expansion program in the postexpansion period, using a negative binomial regression model (to account for the overdispersion of the data) adjusted for the number of clinicians by discipline.

Using the survey data, we computed descriptive statistics (proportion and frequency) on measures of challenges that NHSC clinicians and sites faced in providing SUD and OUD treatment. We tested for significant differences in survey outcomes (2-sided *P* < .05) between rural and nonrural areas using bivariate logistic regression. We used an unadjusted model, since the aim of this study was to describe the prevalence of challenges among NHSC clinicians and sites and differences between rural and nonrural areas, rather than constructing a multivariable model to examine correlates of these challenges. We used the Benjamini-Hochberg method to implement a false discovery rate correction for multiple comparisons.^22^ Since survey responses may be correlated within sites, we also examined the intraclass correlation coefficients for each survey question using a multilevel model. We observed very small intraclass correlation coefficients for the 4 questions in the analysis (0.016, 0.005, 0.004, and 0.001, respectively) and, therefore, did not cluster the SEs at the site level. All analyses were conducted using Stata, version 16 software (StataCorp LLC).

## Results

Table 1 provides the descriptive statics of the clinicians in the claims analysis (1743 in the expansion LRPs and 6085 in the standard LRP) and the survey respondents (3297, including 1304 in rural sites and 1993 in nonrural sites). For the claims analysis, which compared buprenorphine prescribing between clinicians in the expansion programs vs the standard program, the programs included more advanced practice nurses (67.4% [1168] vs 57.6% [3529]), fewer physicians (19.0% [331] vs 23.4% [1400]), fewer physician assistants (13.6% [244] vs 19.1% [1156]), fewer women (75.0% [1307] vs 78.9% [4807]), and fewer individuals with an underrepresented minority status (21.3% [366] vs 29.2% [1765]). The mean (SD) age was 39.4 (8.1) years in the expansion LRPs vs 38.1 (8.4) years in the standard program, and distributions were similar for male (expansion, 418 [24.0%]; standard, 1278 [21.1%]) and nondisclosed (expansion, 18 [1.0%]; standard, 0) gender. In the survey analysis, which compared clinicians in rural and nonrural sites, the rural sites had fewer physicians (13.4% [175] vs 16.2% [322]), fewer dentists (0.6% [9] vs 1.6% [32]), more pharmacists (2.8% [36] vs 0.9% [18]), and fewer individuals with an underrepresented minority status (12.1% [157] vs 26.4% [526]). Age and gender distributions between rural and nonrural respondents were similar (rural: mean [SD] age, 39.9 [9.3] years; 311 men [23.9%], 985 women [75.5%], and 8 who did not disclose [0.6%]; nonrural: mean [SD] age, 39.2 [8.8] years; 460 men [23.1%], 1518 women [76.1%], and 15 who did not disclose [0.8%]).

**Table 1.** Characteristics of the National Health Service Corps Clinicians in the Analytic Sample

Characteristic	Medicaid claims population, %^a^		Survey respondents, %	
	Standard LRP (n = 6085)	Expansion LRPs^b^ (n = 1743)	Nonrural sites (n = 1993)	Rural sites^c^ (n = 1304)
Discipline				
Advanced practice nurse	3529 (57.6)	1168 (67.4)^d^	875 (43.9)	588 (45.1)
Allopathic or osteopathic physician	1400 (23.4)	331 (19.0)^d^	322 (16.2)	175 (13.4)^d^
Physician assistant	1156 (19.1)	244 (13.6)^d^	240 (12.0)	176 (13.5)
Counselor^e^	NA	NA	269 (13.5)	175 (13.4)
Social worker^e^	NA	NA	191 (9.6)	122 (9.4)
Psychologist^e^	NA	NA	38 (1.9)	20 (1.5)
Dentist^e^	NA	NA	32 (1.6)	9 (0.6)^d^
Pharmacist^e^	NA	NA	18 (0.9)	36 (2.8)^d^
Age, mean (SD) [IQR], y^f^	38.1 (8.4) [27.4-49.4]	39.4 (8.1) [28.4-50.4]	39.2 (8.8) [33.0-44.0]	39.9 (9.3) [33.0-46.0]
Gender^f^				
Female	4807 (78.9)	1307 (75.0)^d^	1518 (76.1)	985 (75.5)
Male	1278 (21.1)	418 (24.0)	460 (23.1)	311 (23.9)
Did not disclose	0	18 (1.0)	15 (0.8)	8 (0.6)
Underrepresented minority^f^^,^^g^	1765 (29.2)	366 (21.3)^d^	526 (26.4)	157 (12.1)^d^

Abbreviations: LRP, Loan Repayment Program; NA, not applicable.

^a^ Clinicians who prescribed buprenorphine for opioid use disorder.

^b^ The expansion LRPs are the Substance Use Disorder and Rural Community LRPs.

^c^ Rural area is defined by the Health Resources and Services Administration Federal Office of Rural Health Policy.

^d^ Indicates significant differences at *P* < .05.

^e^ Counselors, social workers, and psychologists cannot prescribe buprenorphine and thus were excluded from the claims analyses. Dentists and pharmacists can prescribe, but they were not eligible for the expansion programs. Therefore, to make the populations eligible for the claims analysis comparable, dentists and pharmacists in the expansion programs were also excluded from the claims analyses.

^f^ Age, gender, and underrepresented minority status are self-reported in National Health Service Corps application data (fiscal years 2017-2021).

^g^ Underrepresented minority group status includes persons from the following backgrounds: American Indian or Alaska Native, Black or African American, Native Hawaiian or Other Pacific Islander, and Hispanic (all races), as defined by the Health Resources and Services Administration.^15^

Table 2 reports the percentage and total number of beneficiaries who filled at least 1 prescription for the standard and expansion LRPs in the preexpansion and postexpansion periods for individuals with OUD overall, individuals with OUD living in rural areas, and individuals with OUD living in areas with a high SVI score. The preexpanison period reflects claims data from NHSC clinicians who were already working in the standard program or who later entered an expansion program. There was substantial growth in the percentage of beneficiaries who filled prescriptions in both the standard and expansion LRPs, but the growth was significantly larger among clinicians in the expansion LRPs (difference in differences, 7175.5; 95% CI, 4895.7-9455.3; *P* < .001) (eTable 2 in Supplement 1 shows the regression results). During the 2.5 years following the expansion, the percentage of beneficiaries with OUD who filled a prescription increased from 18.9% to 43.7%, or 123 422 additional beneficiaries. Of this increase, 73.0% was attributed to NHSC clinicians in the expansion programs (90 040 beneficiaries). An additional 45 523 beneficiaries with OUD living in rural areas (an increase from 20.8% to 55.7%) and an additional 31 964 beneficiaries with OUD (an increase from 17.0% to 36.7%) living in areas with a high SVI score filled prescriptions in the postexpansion period. Of beneficiaries living in rural areas and areas with a high SVI score, 71.0% filled prescriptions from clinicians in the expansion LRPs in the postexpansion period.

**Table 2.** Medicaid Beneficiaries With a Filled Prescription for Buprenorphine, Before and After the National Health Service Corps Program Expansion^a^

Category	No. of beneficiaries filling at least 1 prescription (%)				Total overall increase (postexpansion − preexpansion), No.	*P* value for differential increase for expansion LRP^b^
	Preexpansion (January 1, 2017, to June 30, 2019)		Postexpansion (July 1, 2019, to December 31, 2021)			
	Standard LRP	Expansion LRPs	Standard LRP	Expansion LRPs		
OUD diagnosis^c^	9623 (7.4)	30 477 (36.6)	43 005 (18.9)	120 517 (82.2)	123 422	<.001
OUD diagnosis, living in rural areas^d^	3149 (9.1)	8985 (37.5)	16 805 (28.3)	40 852 (92.6)	45 523	<.001
OUD diagnosis, living in high SVI areas^e^	2867 (6.8)	7827 (38.9)	12 271 (16.0)	30 387 (77.3)	31 964	<.001

Abbreviations: LRP, Loan Repayment Program; OUD, opioid use disorder; SVI, Social Vulnerability Index.

^a^ Categories shown are not mutually exclusive. Data source is Medicaid prescription claims data and Health Resources and Services Administration administrative data.

^b^ Significant differences in the change between the standard and expansion LRPs, pre- and postexpansion period, using a negative binomial regression model adjusted for the number of clinicians by discipline. Total aggregated from unique patient counts during five 6-month periods in the preexpansion and postexpansion period. Beneficiaries could be counted in more than one 6-month period.

^c^ Opioid use disorder diagnosis in the past 12 months.

^d^ Rural areas were defined using the Health Resources and Services Administration Federal Office of Rural Health Policy.

^e^ Data from the Centers for Disease Control and Prevention Agency for Toxic Substances and Disease Registry.

Table 3 reports challenges to SUD and OUD treatment for clinicians and sites in rural and nonrural areas. The greatest challenge among NHSC clinicians providing SUD treatment was limited treatment resources, such as referrals to counselors and detoxification programs (2108 of 3113 [67.7%; 95% CI, 65.6%-69.7%]); this challenge was significantly higher for rural compared with nonrural clinicians (964 of 1303 [74.0%; 95% CI, 70.8%-76.9%] vs 1144 of 1810 [63.2%; 95% CI, 60.4%-66.0%], a 10.8–percentage point difference). Limited treatment resources was the second most common challenge reported among NHSC sites (1997 of 4732 [42.1%; 95% CI, 40.7%-43.5%]); this challenge was also significantly higher among rural sites compared with nonrural sites (1164 of 2498 [46.6%; 95% CI, 39.0%-48.5%] vs 827 of 2234 [37.0%; 95% CI, 35.0%-39.0%], a 9.6–percentage point difference). A lack of trained staff was the second most common challenge reported by clinicians (1725 of 3113 [55.4%; 95% CI, 53.2%-57.6%]) and the greatest challenge reported by sites (2206 of 4732 [46.5%; 95% CI, 45.1%-48.0%]), with higher rates among clinicians (though not significantly, after adjusting for multiple comparisons) and sites in rural areas compared with nonrural areas (4.0 percentage points higher among rural sites). Limited access to OUD treatment options was reported by 1286 of 3113 clinicians (41.3%; 95% CI, 39.1%-43.4%) and was 11.4 percentage points higher among rural clinicians compared with nonrural clinicians. In addition, 968 of 4732 sites (20.4%; 95% CI, 19.3%-21.6%) reported limited OUD treatment options, with a significantly higher rate among rural sites vs nonrural sites (607 of 2498 [24.3%; 95% CI, 17.7%-26.1%] vs 362 of 2234 [16.2%; 95% CI, 14.7%-17.7%], an 8.1–percentage point difference).

**Table 3.** Challenges With SUD Treatment

Which specific challenges do you face in providing SUD treatment services at your NHSC site?^a^	No. (%) [95% CI]^b^			*P* value
	Nonrural	Rural	Overall	
**NHSC clinicians**				
No. of clinicians	1810	1303	3113	NA
Lack of routine screening for SUD	391 (21.6) [19.3-24.1]	281 (21.6) [18.9-24.5]	672 (21.6) [19.8-23.5]	>.99
Limited treatment resources (eg, referrals to counselors, detoxification)	1144 (63.2) [60.4-66.0]	964 (74.0) [70.8-76.9]^c^	2108 (67.7) [65.6-69.7]	<.001
Limited capacity to provide telehealth for SUD	288 (15.9) [13.9-18.2]	228 (17.5) [15.0-20.2]	517 (16.6) [15.0-18.3]	.36
Limited time for 1-on-1 services	585 (32.3) [29.6-35.1]	401 (30.8) [27.8-34.1]	987 (31.7) [29.6-33.8]	.50
Limited number of trained staff	965 (53.3) [50.3-56.2]	761 (58.4) [54.9-61.7]	1725 (55.4) [53.2-57.6]	.03
Limited space or poor infrastructure	378 (20.9) [18.6-23.4]	274 (21.0) [18.3-23.9]	651 (20.9) [19.2-22.8]	.96
Limited integration or coordination with primary care	453 (25) [22.5-27.6]	309 (23.7) [20.9-26.7]	760 (24.4) [22.6-26.4]	.51
Limited access to OUD options	661 (36.5) [33.8-39.4]	624 (47.9) [44.5-51.3]^c^	1286 (41.3) [39.1-43.4]	<.001
Patients report they cannot afford the cost	521 (28.8) [26.2-31.5]	413 (31.7) [28.6-34.9]	934 (30) [28.0-32.1]	.18
Patient factors that affect adherence	903 (49.9) [47.0-52.8]	644 (49.4) [46.0-52.9]	1547 (49.7) [47.5-51.9]	.84
Insufficient team-based care	360 (19.9) [17.6-22.3]	220 (16.9) [14.5-19.6]	579 (18.6) [16.9-20.4]	.09
Other	42 (2.3) [1.6-3.4]	39 (3.0) [2.0-4.4]	81 (2.6) [2.0-3.4]	.41
No challenges	33 (1.8) [1.2-2.7]	20 (1.5) [0.9-2.7]	53 (1.7) [1.2-2.4]	.64
**NHSC sites**				
No. of sites	2234	2498	4732	NA
Lack of routine screening for SUD	168 (7.5) [6.5-8.6]	200 (8.0) [8.6-9.1]	370 (7.8) [7.0-8.6]	.53
Limited treatment resources (eg, referrals to counselors, detoxification)	827 (37.0) [35.0-39.0]	1164 (46.6) [39.0-48.5]^c^	1997 (42.1) [40.7-43.5]	<.001
Limited capacity to provide telehealth for SUD	252 (11.3) [10.1-12.7]	345 (13.8) [12.7-15.2]^c^	598 (12.6) [11.7-13.6]	.01
Limited time for 1-on-1 services	308 (13.8) [12.4-15.3]	395 (15.8) [15.3-17.3]^c^	702 (14.8) [13.8-15.9]	.050
Limited number of trained staff	994 (44.5) [42.5-46.6]	1212 (48.5) [46.6-50.4]^c^	2206 (46.5) [45.1-48.0]	.007
Limited space or poor infrastructure	362 (16.2) [14.7-17.8]	417 (16.7) [17.8-18.2]	783 (16.5) [15.5-17.6]	.62
Limited integration or coordination with primary care	203 (9.1) [8.0-10.4]	275 (11) [10.4-12.3]^c^	479 (10.1) [9.3-11.0]	.04
Limited access to OUD treatment options	362 (16.2) [14.7-17.7]	607 (24.3) [17.7-26.1]^c^	968 (20.4) [19.3-21.6]	<.001
Insufficient team-based care	130 (5.8) [4.9-6.8]	165 (6.6) [6.8-7.7]	294 (6.2) [5.6-6.9]	.21
Patients report they cannot afford the cost	266 (11.9) [10.4-13.7]	360 (14.4) [13.7-16.2]^c^	626 (13.2) [12.1-14.4]	.04
Patient factors that affect adherence	572 (25.6) [23.8-27.5]	662 (26.5) [27.5-28.3]	1238 (26.1) [24.8-27.3]	.49
Other	235 (10.5) [9.3-11.8]	235 (9.4) [11.8-10.6]	470 (9.9) [9.0-10.7]	.20
No challenges	179 (8.0) [6.9-9.2]	120 (4.8) [9.2-5.8]^c^	299 (6.3) [5.7-7.1]	<.001

Abbreviations: NA, not applicable; NHSC, National Health Service Corps; OUD, opioid use disorder; SUD, substance use disorder.

^a^ From the NHSC clinician and site surveys, 2020-2022. This question was asked only if respondents reported any challenges to providing SUD treatment and excluded missing values (missing for 20 clinicians and 1265 sites).

^b^ Column percentages do not sum to 100 because respondents could select more than 1 answer.

^c^ Indicates a significant difference between rural and nonrural clinicians or rural and nonrural sites at *P* < .05. Rural and nonrural (defined using the Health Resources and Services Administration Federal Office of Rural Health Policy) results were compared using logistic regression.

Respondents who reported limited access to OUD treatment options as a challenge were asked which specific services were lacking or limited at their site (Table 4). The top responses for both clinicians and sites were addiction counseling (882 of 1254 clinicians [70.3%; 95% CI, 67.0%-73.4%] and 566 of 937 sites [60.3%; 95% CI, 57.2%-63.4%]) and access to MOUD (780 of 1254 clinicians [62.2%; 95% CI, 58.7%-65.4%] and 637 of 937 sites [67.9%; 95% CI, 64.8%-70.8%]). We did not find significant differences between rural and nonrural clinicians for these outcomes. Significantly more rural NHSC sites compared with nonrural sites reported a lack of addiction counseling (378 of 591 [64.0%; 95% CI, 59.5%-67.7%] vs 188 of 346 [54.3%; 95% CI, 49.0%-59.5%], a 9.7–percentage point difference) and diagnosis by licensed professionals (371 of 591 [62.8%; 95% CI, 59.8%-66.6%] vs 189 of 346 [54.6%; 95% CI, 49.3%-59.8%], an 8.2–percentage point difference).

**Table 4.** Challenges With Opioid Use Disorder Treatment

Limited access to opioid use disorder treatment options is a challenge. Which services are limited?^a^	No. (%) [95% CI]			***P* value** ^b^
	Nonrural	Rural	Overall	
**NHSC clinicians**				
No. of clinicians	640	614	1254	NA
Diagnosis by a licensed professional	359 (56.1) [51.2-61.0]	364 (59.3) [54.3-64.2]	724 (57.7) [54.2-61.1]	.37
Addiction counseling	435 (68.0) [63.3-72.4]	446 (72.7) [68.0-77.0]	882 (70.3) [67.0-73.4]	.15
Medication-assisted treatment (ie, buprenorphine, methadone, naltrexone)	398 (62.2) [57.4-66.9]	381 (62.1) [57.2-66.7]	780 (62.2) [58.7-65.4]	.96
Other: please specify	54 (8.5) [6.2-11.6]	54 (8.8) [6.3-12.1]	108 (8.6) [6.9-10.8]	.90
**NHSC sites**				
No. of sites	346	591	937	NA
Diagnosis by a licensed professional	189 (54.6) [49.3-59.8]	371 (62.8) [59.8-66.6]^c^	561 (59.8) [56.6-62.9]	.02
Addiction counseling	188 (54.3) [49.0-59.5]	378 (64.0) [59.5-67.7]^c^	566 (60.3) [57.2-63.4]	.004
Medication-assisted treatment	235 (67.9) [62.8-72.6]	401 (67.9) [72.6-71.5]	637 (67.9) [64.8-70.8]	.98
Other: please specify	42 (12.1) [9.1-16.0]	55 (9.3) [16.0-11.9]	97 (10.3) [8.5-12.5]	.18

Abbreviations: NA, not applicable; NHSC, National Health Service Corps.

^a^ From the NHSC clinician and site surveys, 2020-2022. This question was asked among clinicians and sites who answered affirmatively to limited access to the opioid use disorder treatment options shown in Table 3. Analysis excludes missing values (missing for 34 clinicians and 25 sites).

^b^ Rural and nonrural (defined using the Health Resources and Services Administration Federal Office of Rural Health Policy) results were compared using logistic regression.

^c^ Indicates a significant difference between rural and nonrural clinicians or sites at *P* < .05.

Table 5 shows survey results for challenges to prescribing buprenorphine among clinicians eligible to prescribe and reasons why they did not prescribe. We did not find significant differences between clinicians working in rural and nonrural areas. The greatest challenge was concern over medication diversion or misuse (929 of 2140 [43.4%; 95% CI, 40.8%-46.1%]), while the second greatest challenge was a lack of mental health services to complement MOUD (773 of 2140 [36.1%; 95% CI, 33.6%-38.6%]). Lack of supervision and the ability to consult peers on prescribing as a challenge was reported by 400 of 2140 clinicians (18.7%; 95% CI, 16.7-20.9).

**Table 5.** Challenges to and Reasons for Not Prescribing Buprenorphine Among NHSC Clinicians

Question	No. (%) [95% CI]^a^			***P* value** ^b^
	Nonrural	Rural	Overall	
**Challenges in prescribing buprenorphine**				
Do you encounter any of the following challenges in prescribing buprenorphine?^c^				
No. of respondents	1340	800	2140	NA
Lack of eligible patients	169 (12.6) [10.5-15.0]	101 (12.6) [9.9-15.7]	270 (12.6) [10.9-14.4]	.99
Eligible patients cannot afford it	155 (11.6) [9.7-14.0]	121 (15.1) [12.2-18.4]	276 (12.9) [11.2-14.8]	.07
Lack of mental health services to complement medication use	470 (35.1) [31.9-38.4]	302 (37.7) [33.6-42.0]	773 (36.1) [33.6-38.6]	.33
Lack of supervision, mentorship, specialist backups, peer consultation	235 (17.5) [15.1-20.2]	166 (20.7) [17.4-24.4]	400 (18.7) [16.7-20.9]	.15
Lack of capacity to treat patients with OUD	182 (13.6) [11.4-16.1]	94 (11.7) [9.2-14.8]	276 (12.9) [11.2-14.8]	.32
Compliance with Drug Enforcement Administration instructions	25 (1.9) [1.2-3.1]	25 (3.1) [1.9-5.0]	51 (2.4) [1.7-3.3]	.17
Concern about medication diversion or misuse	556 (41.5) [38.2-44.8]	374 (46.7) [42.4-51.0]	929 (43.4) [40.8-46.1]	.06
Other	88 (6.6) [5.1-8.5]	59 (7.4) [5.4-10.0]	148 (6.9) [5.6-8.4]	.59
No challenges	268 (20.0) [17.5-22.8]	130 (16.3) [13.4-19.8]	398 (18.6) [16.7-20.8]	.09
**Reasons that NHSC clinicians do not prescribe buprenorphine**				
What are the reasons that you do not prescribe buprenorphine?^c^				
No. of respondents	663	369	1032	NA
Lack of eligible patients	108 (16.3) [13.1-20.3]	71 (19.3) [14.6-24.9]	180 (17.4) [14.6-20.6]	.35
Eligible patients cannot afford it	19 (2.9) [1.7-5.1]	5 (1.3) [0.4-4.0]	24 (2.3) [1.4-3.9]	.22
Lack of other mental health services to complement medication use	83 (12.5) [9.6-16.1]	69 (18.6) [14.0-24.2]^b^	152 (14.7) [12.1-17.7]	.04
Lack of supervision, mentorship, specialist backups, or peer consultation	190 (28.6) [24.4-33.2]	100 (27.0) [21.6-33.2]	290 (28.1) [24.7-31.7]	.67
Lack of capacity to treat patients with OUD	134 (20.2) [16.5-24.4]	79 (21.5) [16.6-27.3]	213 (20.6) [17.6-24.0]	.70
Compliance with Drug Enforcement Administration instructions	13 (1.9) [1.0-3.8]	11 (3.1) [1.5-6.3]	24 (2.3) [1.4-3.9]	.37
Concern about medication diversion or misuse	70 (10.5) [7.9-13.9]	41 (11.1) [7.6-15.9]	110 (10.7) [8.5-13.4]	.83
Organizational factors (eg, prescribing policies, zero tolerance for continued drug use)	169 (25.5) [21.5-30.0]	100 (27.2) [21.7-33.4]	269 (26.1) [22.8-29.7]	.65
Not in scope of the current role	221 (33.4) [28.9-38.2]	114 (30.9) [25.2-37.2]	335 (32.5) [28.9-36.3]	.53
Other	60 (9.0) [6.6-12.2]	19 (5.2) [3.0-9.0]	78 (7.6) [5.8-10.0]	.09

Abbreviations: NA, not applicable; NHSC, National Health Services Corps; OUD, opioid use disorder.

^a^ Column percentages may not sum to 100 because respondents could select more than 1 answer.

^b^ Rural and nonrural (defined using the Health Resources and Services Administration Federal Office of Rural Health Policy) results were compared using a logistic regression.

^c^ From the NHSC Clinician Survey, 2020-2022. The question on challenges to prescribing was asked only among clinicians who reported prescribing buprenorphine. The question on reasons for not prescribing was only asked if clinicians reported that they did not prescribe buprenorphine in a previous question. The analysis excludes missing values (missing for 34 clinicians for the first question and for 8 clinicians for the second question).

A total of 1032 clinicians indicated they did not prescribe buprenorphine, even though it was within the scope of their current role. The most common reason given by both rural and nonrural clinicians was a lack of supervision, mentorship, or peer consultation (290 [28.1%; 95% CI, 24.7%-31.7%]). Organizational factors (eg, policies around initiation of treatment and a zero tolerance for continued drug use) were the second most common reason for not prescribing among all clinicians (269 [26.1%; 95% CI, 22.8%-29.7%]). Clinicians working in rural areas were significantly more likely than nonrural clinicians to report a lack of other mental health services to complement medication (69 of 369 [18.6%; 95% CI, 14.0%-24.2%] vs 83 of 663 [12.5%; 95% CI, 9.6%-16.1%], a 6.1–percentage point difference).

## Discussion

This cross-sectional study found that the NHSC LRP expansion was associated with an increase in the percentage of Medicaid beneficiaries with OUD who were prescribed buprenorphine. More than 3 times as many beneficiaries overall, and 3.8 times as many beneficiaries in rural areas, received a prescription in the 2.5-year period following the expansion than in the preceding 2.5-year period. Clinicians reached more beneficiaries in rural areas and areas with high SVI scores, indicating that the NHSC program expansion contributed to the recruitment and placement of clinicians in high-need areas.

However, our findings show that continued expansion in OUD treatment may be hampered by inadequate counseling resources to support MOUD, a lack of trained staff, and limited availability of peers or supervisors to assist clinicians in prescribing and treating OUD. Among the clinicians who reported limited access to OUD treatment options, patient access to addiction counseling was the largest barrier reported by clinicians (70.3%). Just over one-third (36.1%) of clinicians reported that a lack of mental health services to complement medication use was the greatest challenge to OUD treatment and a reason expressed by 1 in 8 clinicians (14.7%) for not prescribing MOUD. Our results reflect survey data collected during the COVID-19 pandemic when the health care workforce faced substantial attrition and burnout; however, the high rates of respondents indicating limited personnel to support MOUD suggest the need to continue to train and retain behavioral health care workers in underserved areas.^23^ For example, HRSA estimates that since 2021, demand for behavioral health clinicians (ie, addiction counselors, psychiatrists, social workers, and therapists) has increased by 8%, and supply has decreased by 2%.^24^ This workforce shortage may stem from a long history of regulatory constraints on clinician capacity to prescribe MOUD, including the X-waiver’s requirements regarding the clinician’s training hours and number of patients. However, the removal of the waiver does not address the challenges to team-based OUD treatment observed in this study, limited addiction training during medical education, and stigma around the effectiveness of MOUD.^23^

Strategies to expand MOUD delivery require an adequate supply of behavioral health clinicians and other nonprescribing health care workers to help patients transition across settings.^25^ Federal programs that expand the number of behavioral health clinicians and create incentives to work in underserved areas, including the NHSC LRPs and HRSA grant programs such as the Behavioral Health Workforce Education and Training program, could play an essential role in building and retaining the nation’s behavioral health workforce.

### Limitations

This study has several limitations. The significant increase in the number of Medicaid beneficiaries with OUD who had a prescription for buprenorphine cannot be attributed solely to NHSC expansion, as during the study period, both federal and state policies were enacted to improve access to buprenorphine, including enabling telehealth for prescribing and increasing patient panel sizes, and studies have shown that these changes had some effect.^26,27^ In addition, our findings are not stratified at the state level, and state-level policies and guidelines can facilitate or limit prescribing. Medicaid data quality also varies by state, and claims data only reflect filled prescriptions. Because of incomplete claims, missing prescriber NPIs, or unfilled prescriptions, the results may undercount all prescriptions written by NHSC clinicians. We cannot quantify the number of unfilled prescriptions, and we were not able to locate prior research that examined what percentage of prescriptions for buprenorphine remain unfilled among Medicaid beneficiaries. The study was conducted before the removal of the X-waiver in December 2022; thus, future research should continue to study the persistence of challenges to prescribing and comprehensive treatment, as well as the variation in challenges across states, as policies continue to evolve. The findings also reflect results among NHSC clinicians and sites and may not be generalizable to the challenges faced by clinicians who do not work in underserved areas. While it may be possible that some NHSC clinicians were newly licensed recent graduates and did not prescribe buprenorphine before they entered the NHSC LRP, the mean age of the prescribing clinicians was 38 years, which suggests that most were not recent graduates. One reason for discrepancies between NHSC clinician and site reports of perceived challenges is that NHSC sites may have more than 1 facility, and site respondents were asked to summarize their responses across all facilities.

## Conclusions

This cross-sectional study found that the LRP expansion was associated with increases in access to buprenorphine. These findings also suggest that clinicians and care teams may need continued training, mentorship, staffing, and connections to referral networks to provide appropriate, comprehensive SUD and OUD treatment.

## Notes

Supplement 1. **eTable 1.** Descriptive Characteristics for Clinicians in the Survey Analysis, by Standard and Expansion Programs **eTable 2.** Regression-Adjusted Total Number of Beneficiaries Who Filled a Prescription for Buprenorphine From National Health Service Corps Clinicians, Pre and Post Expansion, by Loan Repayment Program Group and Beneficiary Characteristic **eMethods 1.** Diagnosis Codes **eMethods 2.** Medicaid Data Quality **eMethods 3.** Survey Questionnaires, Response Rates, Methodology

Supplement 2.
Nonauthor Collaborators

Supplement 3.
Data Sharing Statement
