# Supplementary material for: Buprenorphine Prescribing and Challenges Faced Among National Health Service Corps Clinicians
Source: JAMA Netw Open. 2024 May 17;7(5):e2411742. doi: 10.1001/jamanetworkopen.2024.11742 (PMC11102013; doi:10.1001/jamanetworkopen.2024.11742)
Supplement: Supplement 1. — eTable 1. Descriptive Characteristics for Clinicians in the Survey Analysis, by Standard and Expansion Programs eTable 2. Regression-Adjusted Total Number of Beneficiaries Who Filled a Prescription for Buprenorphine From National Health Service Corps Clinicians, Pre and Post Expansion, by Loan Repayment Program Group and Beneficiary Characteristic eMethods 1. Diagnosis Codes eMethods 2. Medicaid Data Quality eMethods 3. Survey Questionnaires, Response Rates, Methodology [file jamanetwopen-e2411742-s001.pdf]

Supplemental Online Content

Rowan K, Shah SV, Binns S, et al. Buprenorphine prescribing and challenges faced among National Health Services Corps Clinicians. *JAMA Netw Open*. 2024;7(5):e2411742. doi:10.1001/jamanetworkopen.2024.11742

eTable 1. Descriptive Characteristics for Clinicians in the Survey Analysis, by Standard and Expansion Programs

eTable 2. Regression-Adjusted Total Number of Beneficiaries Who Filled a Prescription for Buprenorphine From National Health Service Corps Clinicians, Pre and Post Expansion, by Loan Repayment Program Group and Beneficiary Characteristic

eMethods 1. Diagnosis Codes

eMethods 2. Medicaid Data Quality

eMethods 3. Survey Questionnaires, Response Rates, Methodology

This supplemental material has been provided by the authors to give readers additional information about their work.

© 2024 Rowan K et al. *JAMA Network Open.*

eTable 1. Descriptive Characteristics for Clinicians in the Survey Analysis, by Standard and Expansion Programs

**Discipline**	**Standard LRP (%) (n=1,907)**	**Expansion LRPs (%) (n=1,390)**
Advanced Practice Nurse	43.9	45.0
Allopathic or Osteopathic Physician	15.8	14.1
Physician Assistant	15.1	9.2*
Counselor	9.5	18.9*
Social Worker	11.0	7.5*
Psychologist	2.0	1.4
Dentist	2.1	NA
Pharmacist	NA	3.9
**Age (mean, standard deviation, inter-quartile range)**	39.1 (9.0) (IQR=13)	39.9 (9.0) (IQR=12)
**Female**	77.9	74.5*
**Underrepresented Minority**	22.3	18.7*

Notes: NHSC Clinician application data (fiscal years 2019-2021). LRP= Loan Repayment Program. The expansion LRPs are the Substance Use Disorder and Rural Community LRPs. NA=these disciplines were not eligible to participate in the respective loan repayment program. Age, gender, rural background, and underrepresented minority status are self-reported in NHSC application data. Underrepresented minority status includes persons from the following backgrounds: American Indian or Alaska Native, Black or African American, Native Hawaiian or Other Pacific Islander, and Hispanic (all races). See the HRSA Glossary (https://bhw.hrsa.gov/glossary#u) for a complete description.

© 2024 Rowan K et al. *JAMA Network Open.*

eTable 2. Regression-Adjusted Total Number of Beneficiaries Who Filled a Prescription for Buprenorphine From National Health Service Corps Clinicians, Pre and Post Expansion, by Loan Repayment Program Group and Beneficiary Characteristic

Modeled results	Standard LRP	Expansion LRPs	Difference in differences
	Pre-post difference	Pre-post difference	
**Beneficiaries with OUD who filled a prescription (a)**			
Estimate	32887.8	104159.3	7175.5
P-value	<0.001	<0.001	<0.001
Lower 95% confidence interval	32422.3	101018.9	4895.7
Upper 95% confidence interval	33353.3	107299.6	9455.3
**Beneficiaries living in rural areas who filled a prescription (b)**			
Estimate	13396.1	38223.0	3010.6
P-value	<0.001	<0.001	<0.001
Lower 95% confidence interval	13216.2	37092.5	2261.6
Upper 95% confidence interval	13576.0	39353.4	3759.7
**Beneficiaries living in high social vulnerability who filled a prescription (c)**			
Estimate	9291.6	25366.5	1327.4
P-value	<0.001	<0.001	<0.001
Lower 95% confidence interval	9158.2	24598.4	800.8
Upper 95% confidence interval	9425.1	26134.6	1854.0

Notes: Results were compared for the pre- and post-period using a negative binomial regression model of the effect of time on the outcome adjusted for the number of clinicians in each discipline. The table shows the modeled marginal effects and confidence intervals, and associated P-values for a test of significant differences at P<.05 within each LRP and the difference-in-differences pre- and post-expansion. The pre-period was January 1, 2017 to June 30, 2019, and the post-period was July 1, 2019 to December 31, 2021. LRP=Loan Repayment Program.

Sources: Medicaid claims are from the Transformed Medicaid Statistical Information System. Clinician program data are from Health Resources and Services Administration. (a)=opioid use disorder (OUD) diagnosis in the past 12 months. (b)=Rural areas were defined using Federal Office of Rural Health Policy (FORHP) data. (c)= Social Vulnerability Index data from the Centers for Disease Control and Prevention Agency for Toxic Substances and Disease Registry (CDC/ATSDR).

© 2024 Rowan K et al. *JAMA Network Open.*

eMethods 1. Diagnosis Codes. International Classification of Diseases 10 Clinical Modification (ICD-10-CM) Diagnosis Codes for Opioid Use Disorder:

F1110, F11120, F11121, F11122, F11129, F1113, F1114, F11150, F11151, F11159, F11181, F11182, F11188, F1119, F1120, F11220, F11221, F11222, F11229, F1123, F1124, F11250, F11251, F11259, F11281, F11282, F11288, and F1129.

eMethods 2. Medicaid Claims Data Quality Summary

Claims Volume	High Concern	Unusable
2017	Florida, North Carolina	None
2018	Ohio, North Carolina	None
2019	North Carolina	None
2020	North Carolina	None
2021	North Carolina, Utah	None
**Missing Prescribing NPI**		
2017	Maine	Florida
2018	Maine	Florida
2019	Maine, New Hampshire	Florida
2020	Maine, New Hampshire	Florida
2021	Maine	Florida

Source: Medicaid Data Quality Atlas. https://www.medicaid.gov/dq-atlas/landing/topics/single/table?topic=g16m48&tafVersionId=25

eMethods 3. Survey Questionnaires, Response Rates, Methodology

Link to Office of Management and Budget Information Collection Review, Control No: 0906-0054
https://www.reginfo.gov/public/do/DownloadDocument?objectID=100715901
Link to online NHSC Clinician Survey Questionnaire:
https://www.norc.org/content/dam/norc-org/pdfs/NHSC%20Clinician%20Survey.pdf
Link to online NHSC Site Survey Questionnaire:
https://www.norc.org/content/dam/norc-org/pdfs/NHSC%20Site%20Survey%202021.pdf

Survey Response Rates

Survey Year	Total # of Respondents/Total Population	Response rate
**NHSC Clinician**		
2020	2,688/4,012	67.0
2021	3,514/ 5,956	59.0
2022	3,783/ 6,282	60.3
**NHSC Sites**		
2020	2,017/3,707	54.4
2021	2,071/4,344	47.4
2022	1,921/ 4,169	46.1

Notes: the total number of respondents is the overall response rate and larger than the total number who responded to the specific questions in the analyses.

© 2024 Rowan K et al. *JAMA Network Open.*

IRB Approval: NORC's Institutional Review Board (IRB) reviewed all aspects of the study's data collection. The study's activities were determined not to be human subjects research by the NORC IRB (IRB00000967), under its Federal Assurance #FWA00000142.

Survey Methods. Survey instruments were developed using existing survey questions available in the public domain and previous surveys conducted by HRSA or designed by NORC and finalized in consultation with HRSA. Instruments were reviewed through an iterative process that included internal and external survey methodologists and subject matter experts in SUD treatment, health care workforce research, and statistics, and were pilot-tested with six clinicians and six sites.

The complete populations and email contact information for NHSC clinicians and sites were obtained from HRSA administrative data. Each instrument was administered via the web to the census population of all units of analysis (i.e., all clinicians and sites). Surveys were administered on a staggered basis beginning in early September of each year and lasted approximately eight weeks. For NHSC sites, we surveyed one main point of contact (POC) per site. Because NHSC sites may have multiple facilities, we asked respondents to consider all facilities at their site when answering survey questions. Thus, an NHSC "site" respondent may be answering on behalf of one or more facilities.

Survey outreach and fielding: NORC emailed an advance letter announcing the survey, highlighting the Bureau of Health Workforce sponsorship of the survey, and describing the importance of the data being collected. We then sent an invitation email containing a secure link to the survey. Throughout data collection, respondents were encouraged to follow up with project staff via a dedicated project email and toll-free number with any questions about the surveys. We also provided a project web page on the NORC website that included summary information about the evaluation, Frequently Asked Questions about the surveys, and electronic versions of each instrument for reference. After the start of the survey, we sent weekly email reminders, mailed letter reminders, and a one-time text to clinicians to follow up with respondents who had not completed the survey.

NHSC Clinician survey weighting. We examined nonresponse by program type in the NHCS clinician file and found statistically significant differences at ($P<.05$) in response rates across programs. Weights were then created to adjust for clinician nonresponse by post-stratifying on program type; the weights allowed survey results to reflect the distribution of the overall LRP population by program participation.

© 2024 Rowan K et al. *JAMA Network Open.*
