# Supplementary material for: Buprenorphine Prescribing and Challenges Faced Among National Health Service Corps Clinicians
Source: JAMA Netw Open. 2024 May 17;7(5):e2411742. doi: 10.1001/jamanetworkopen.2024.11742 (PMC11102013; doi:10.1001/jamanetworkopen.2024.11742)
Supplement: Supplement 2. — Nonauthor Collaborators [file jamanetwopen-e2411742-s002.pdf]

Supplemental Online Content: Nonauthor Collaborators
*First name, last name, and suffix (if applicable) are required and will appear in PubMed.

*Group Name(s): Bureau of Health Workforce Substance Use Disorder Evaluation Team							
***First Name and Middle Initial(s)**	***Last Name**	***Suffix (eg, Jr, III)**	Academic Degrees	Institution	Location (city, state/province, country)	Role or Contribution, eg, chair, principal investigator	Group (if more than 1 Group listed in the byline) and/or Subgroup (eg, Steering Committee)
Srabani	Das	none	MS	NORC at the University of Chicago	Bethesda, Maryland, USA	survey fielding	
Kiplin	Kaldahl	none	MS	NORC at the University of Chicago	Fort Collins, Colorado, USA	survey fielding	
Ryan	Murphy	none	MPH	NORC at the University of Chicago	Chicago, Illinois, USA	claims analysis	
