# Supplementary material for: Buprenorphine Prescribing and Challenges Faced Among National Health Service Corps Clinicians
Source: JAMA Netw Open. 2024 May 17;7(5):e2411742. doi: 10.1001/jamanetworkopen.2024.11742 (PMC11102013; doi:10.1001/jamanetworkopen.2024.11742)
Supplement: Supplement 3. — Data Sharing Statement [file jamanetwopen-e2411742-s003.pdf]

Data Sharing Statement

Rowan. Buprenorphine Prescribing and Challenges Faced Among National Health Service Corps Clinicians. *JAMA Netw Open*. Published May 17, 2024.
doi:10.1001/jamanetworkopen.2024.11742

Data
Data available: No

Additional Information
Explanation for why data not available: Medicaid claims data were secured through a Data Use Agreement and only available through Center for Medicare and Medicaid Virtual Research Data Center. De-identified survey and administrative data are unavailable due to privacy laws, but aggregated data dashboards on National Health Service Corps applicants, clinicians, and alumni are available at https://data.hrsa.gov/data/dashboards.
